# Potential of Nanoparticles Integrated with Antibacterial Properties in Preventing Biofilm and Antibiotic Resistance

**DOI:** 10.3390/antibiotics10111338

**Published:** 2021-11-02

**Authors:** Maheswary Thambirajoo, Manira Maarof, Yogeswaran Lokanathan, Haliza Katas, Nur Fatiha Ghazalli, Yasuhiko Tabata, Mh Busra Fauzi

**Affiliations:** 1 Centre for Tissue Engineering and Regenerative Medicine, Faculty of Medicine, Universiti Kebangsaan Malaysia, Cheras, Kuala Lumpur 56000, Malaysia; mahe_meera@yahoo.com (M.T.); manira@ppukm.ukm.edu.my (M.M.); lyoges@ppukm.ukm.edu.my (Y.L.); 2 Centre for Drug Delivery Research, Faculty of Pharmacy, Universiti Kebangsaan Malaysia, Jalan Raja Muda Abdul Aziz, Kuala Lumpur 50300, Malaysia; haliza.katas@ukm.edu.my; 3 Biomaterials Unit, School of Dental Sciences, Universiti Sains Malaysia, Kota Bharu 16150, Malaysia; fatiha85@usm.my; 4 Department of Biomaterials, Institute for Frontier Medical Sciences, Kyoto University, 53 Kawara-cho Shogoin, Sakyo-ku, Kyoto 606-8507, Japan; yasuhiko@infront.kyoto-u.ac.jp

**Keywords:** nanoparticles, antimicrobial agents, pathogenic microorganisms, biofilm formations, antibiotic resistance, clinical settings

## Abstract

Nanotechnology has become an emerging technology in the medical field and is widely applicable for various clinical applications. The potential use of nanoparticles as antimicrobial agents is greatly explored and taken into consideration as alternative methods to overcome the challenges faced by healthcare workers and patients in preventing infections caused by pathogenic microorganisms. Among microorganisms, bacterial infections remain a major hurdle and are responsible for high morbidity and mortality globally, especially involving those with medical conditions and elderly populations. Over time, these groups are more vulnerable to developing resistance to antibiotics, as bacterial biofilms are difficult to destroy or eliminate via antibiotics; thus, treatment becomes unsuccessful or ineffective. Mostly, bacterial biofilms and other microbes can be found on medical devices and wounds where they disperse their contents which cause infections. To inhibit biofilm formations and overcome antibiotic resistance, antimicrobial-loaded nanoparticles alone or combined with other substances could enhance the bactericidal activity of nanomaterials. This includes killing the pathogens effectively without harming other cells or causing any adverse effects to living cells. This review summarises the mechanisms of actions employed by the different types of nanoparticles which counteract infectious agents in reducing biofilm formation and improve antibiotic therapy for clinical usage.

## 1. Introduction

### Brief Introduction to Nanoparticles

Nanoparticles (NPs) are ultrafine unit particles with one or more dimensions at the nano-size ranging from 1 to 100 nm [1]. There are several factors that influence the physical stability and interactions of NPs with bioactive compounds in vivo. Examples of these factors are the surface morphology, shape, size, and diameter of the particle. The antimicrobial activity of the nanoparticles corresponds to the high surface-area-to-volume ratio. This is because small particles have the largest surface area which increases the interaction with bacteria and improves their antimicrobial activities [2]. In addition, the large surface area to volume also allows the molecules to bind, fuse and integrate the therapeutic agents to the particles [3]. According to Larner and colleagues (2017), NPs often have unexpected visible properties because they are small enough to confine their electrons and produce quantum effects compared to their bulk materials. Thus, NPs confer special optical effects, increase reactivity, and have good stability, but the theory behind these scientific occurrences are still unclear and require further studies for justification [4]. Nanoparticles exhibit unique features and excellent physicochemical characteristics that make them compatible with different biomedical approaches [1,5] including drug delivery systems, radiotherapy, molecular imaging, and treatments for cancer, neurodegenerative disease, lung disease, acquired immune deficiency syndrome (AIDS), and eye disease [6]. Moreover, other industries also highly exploit the use of NPs such as personal care products, electronics devices, buildings, and building materials [7].

**Figure 1:**
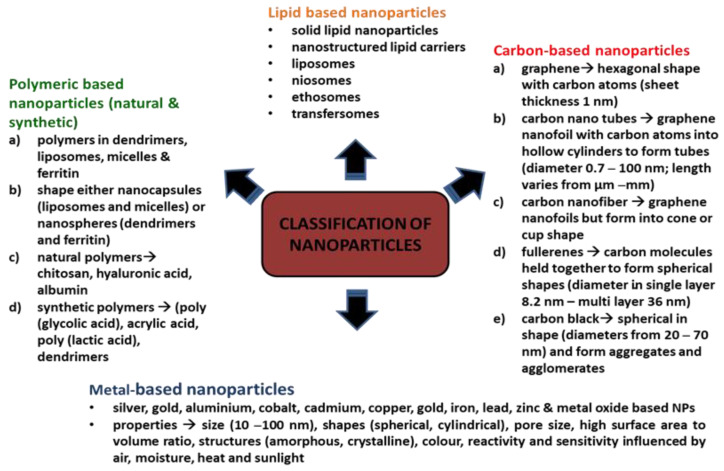
Classifications of nanoparticles.

NPs are classified based on their constituents and dimensional structure. The constituent is the chemical composition of the material, while the dimensional structure refers to 0D, 1D, 2D and 3D. For example, 0D NPs are holospheres, nanolenses and core–shell quantum dots. 1D NPs consist of nanowires, nanotubes, nanorods and many more. 2D NPs are more to multilayers, films, or plates, while 3D is the crystal form of NPs. The classifications are equally important for researchers as guidelines for safe handling, while the performance of the NPs are based on applications [8]. There is a diverse range of NPs that are currently being studied, namely, carbon-based NPs (carbon nanotubes, fullerene and graphene) [8], metallic-based NPs (silver, gold, copper, iron, arsenic, zinc, nickel, chromium, molybdenum, tantalum, cobalt and antimony) [9,10,11], natural polymeric NPs (chitosan, hyaluronic acid and albumin), synthetic polymeric nanoparticles (poly(glycolic acid), acrylic acid and poly(lactic acid) and dendrimers) [12], lipid-based NPs (solid lipid nanoparticles, nanostructured lipid carriers, lipid–drug conjugates and nanoemulsions) [13] and nanocomposites (metal matrix nanocomposites, polymer matrix nanocomposites and ceramic matrix nanocomposites) [14]. Figure 1 depicts NPs based on classifications and properties. Bottom-up and top-down are the two main techniques applied in the synthesis of NPs, whereby these techniques can be biological, physical, and chemical. The biological techniques use microorganisms such as *B. subtilis*, sulphate-reducing bacteria *S. marcescens*, *E. coli* and *B. licheniformis*. Both physical and chemical techniques use different substances, such as ammonia, citrate and sodium borohydride, to synthesise NPs [15]. The bottom-up techniques mainly involve a reduction in chemicals, metal vaporisation, electrodes in a chemical solution, light source, high temperature, precipitation, emulsion, sol-gel and many more. The top-down techniques are based on processing a higher scale of nanoparticles to the simplest forms by using a size reduction technique (ball milling), fraction of the metallic atoms on the surface (metal dispersion techniques), and rapid combustion by electrical coil [16].

NPs have gained attention, particularly in antibiotic therapy, as additional substitutes to treat infections caused by various kinds of microorganisms. NPs have emerged as a new promising treatment to combat bacteria or other microorganisms that have developed resistance to antimicrobial drugs, as NPs have excellent bactericidal or microbicidal effects against microbes, such as bacteria, by associating directly with the bacterial cell wall without perforating the cell [17]. In addition, elevated levels of antimicrobial drugs can be initiated and infused at the infected area through NPs as drug delivery vehicles. These NPs have more absorption capacity (i.e., high bioavailability) with longer half-lives and are less likely to cause cell toxicity [11]. Therefore, incorporating NPs with antimicrobial agents or any active moiety of choice can resolve the microbes’ tolerance to drug treatments, maintain optimal antimicrobial concentration, minimise cytotoxicity effects and act as a novel strategy to eradicate microorganisms that cause diseases [11,17].

## 2. Biofilm Formation and Antibiotic Resistance

It has become a significant challenge for health professionals to treat patients infected by bacteria. This is because prolonged antibiotic or antimicrobial therapies cause bacterial species to become resistant to these treatments since bacteria multiply at a fast rate. The inefficacy of the host immune defence allows for the entry of microbes to colonise and grow [18]. According to Cepas and co-workers (2019), microbes containing adaptable strains or dormant strains, known as “persistent strains”, exhibit antibiotic tolerance and become active once the therapy is withdrawn. These include viruses, bacteria, fungi and parasites. They also added that most of these high-resistance microbe species originated from hospital sites and led to high mortality and morbidity due to the inability of the antibiotic treatments to eliminate these microbes from the infected area. Infections caused by *S. aureus* have affected people in the US at a cost of 4 billion annually for treatment and management [19]. Emerging infectious diseases (EIDs) are a significant burden on global economics and public health. The incidence of EID events is mostly caused by bacterial species encompassing a large number of drug-resistant microbes [20]. Based on the National Institute of Health, the main causative factor that contributes to pathogenic infections in humans is the formation of biofilm, which is accountable for more than 80% of infections [21]. Commonly, biofilms can be found in chronic wounds, renal infections, cystic fibrosis, severe gum infections, inflammation in the endocardium, inflammation of the meninges, medical-device-associated infections, etc. [21,22].

Other than hospital sites, bacterial biofilm is also present on surfaces in nature, pipeline networks and industrial workplaces in which these biofilms play a dual role, either beneficial or detrimental depending on the conditions of the hosts [23,24]. Biofilms are beneficial as biofertilizers to supply nutrients to crop plants, microbial fuel cells to generate electricity, and to clean up contaminated soil and underground water (bioremediation). Therefore, controlling the formation of biofilms is vital to maintaining biofilms for biotechnological processes and to destroying biofilms for preventing microbes from causing diseases and contaminations [25]. Conventionally, there will be more than one type of microbial species embedded within a biofilm such as in the mouth with 500 different bacterial species in a biofilm [24]. Pathogens, particularly bacteria, form biofilms that function as a barrier to growth, multiply, protect against the host immunity system, resist changes in pH and osmotic concentration or resist drug treatments such as multidrug resistance [26,27]. Biofilm is a clump containing a fraction of bacteria encapsulated with an extracellular polymeric matrix or extracellular polymeric substances (EPS) that attach to the surface area [28]. The EPS is in an aqueous environment containing protein substances and plays a role in maintaining the structural integrity of the biofilm, supplying nutrients, supporting adhesion to surfaces and assisting the intercellular signalling molecules, such as cyclic dimeric guanosine monophosphate (c-di-GMP), that are found in bacterial species within the biofilm. In brief, EPS supports colonisation or re-colonisation of bacteria by adhering to surfaces [29].

**Figure 2:**
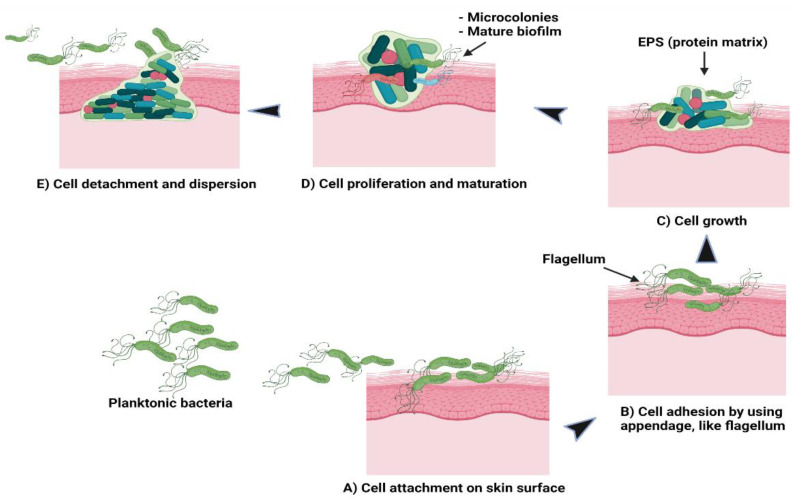
Schematic of (**A**) bacterial cell attachment to the skin’s surface; (**B**) irreversible cell attachment through appendages; (**C**) cells’ growth and EPS synthesis; (**D**) cells’ proliferation and maturation to form microcolonies and biofilms; (**E**) cells’ detachment from biofilms to disperse contents as planktonic cells to form new biofilms.

Theoretically, there are five stages involved in the formation of a bacterial biofilm [30]. In the first stage, the planktonic bacteria reversibly attach to a suitable surface. In the second stage, the cells begin to connect irreversibly through appendages such as fimbriae, pili, flagella, and lipopolysaccharide (LPS). The third stage involves the cells growing and synthesising EPS containing protein matrix. In the fourth stage, the bacterial cells proliferate and mature to form microcolonies and biofilms. At the final stage, some cells detach or are freed from the biofilm and disperse the contents as planktonic cells to form new biofilms in different locations [30,31]. Figure 2 shows the formation of bacterial biofilms. During biofilm maturation, bacterial cells communicate to other cells within the biofilm or with other microbes through a process known as quorum sensing (QS). This helps the bacteria to keep track of their cell numbers and regulate the expression of quorum-specific genes that facilitate bacterial activity including biofilm formation, virulence, cell mobility, extracting nutrients from other cells and deactivation of the immune system [32]. Ideally, *Staphylococcus* species is a group of Gram-positive bacteria with a firm biofilm that causes prolonged infections leading to non-wound healing in chronic wounds [33]. *S. aureus* has the highest resistance followed by *S. epidermidis* towards antibiotics which are primarily used to prevent hospital-acquired infections caused by these species, specifically with implanted devices. Both species form well-established biofilms, causing more infections compared to other Gram-positive or Gram-negative bacteria. In addition to these bacteria, a few examples of bacterial species that can be found in hospital settings are *P. acnes*, *E. faecalis*, *S. viridans*, *E. coli*, *K. pneumoniae*, *P. mirabilis* and *P. aeruginosa* [21,34]. In current practice, patients are given more than one type of antibiotic to reduce infections and resolve problems related to drug resistance. The potency of the drugs depends on the patients’ health conditions and requires clinical examinations, antimicrobial sensitivity pattern and aetiological agents to determine the strain causing the infections [20,35].

Gradually, after prolonged exposure to antibiotics, microbes are able to mutate their genetic material or modify the antimicrobial agents to escape the effects of these drugs and, at the same time, disable the therapeutic functions of the drugs [36]. The mutation takes place in the genome of the bacteria, particularly the mismatch repair system (MMR) that consists of mutS, mutL, mutH, mutT, mutY, mutM and uvrD, whereby these genes together with DNA gyrase and topoisomerase enzymes can elevate the rate of mutation, inhibiting the antimicrobial agents’ activities [37]. The mutation and modification are accomplished with the help of enzymes synthesised by bacteria based on two mechanisms: (i) alteration of the composition of the bacterial enzymes in such a way as to render the ineffectiveness of the antibiotics while retaining the toxic functions of bacteria and (ii) the antibiotics become weak and dysfunctional after the enzymes change the structural parts of the drugs either by modifying or deactivating them. The modification is achieved by adding several bacterial enzyme groups such as phosphate, adenyl and acetyl. These groups can bind to the target sites of the drugs, altering and developing resistance towards the bacterial activities via inactivation through breaking down of the drugs’ hydrolytic action. Moreover, these enzymes are needed by the bacteria in the production of substances for cellular regulatory processes, cell wall components and nucleic acids. Such examples can be seen in the alteration of bacterial ribosomes by the enzyme of 23S rRNA methyltransferases in Gram-positive bacteria and mutation of DNA gyrase in Gram-negative bacteria. In both mechanisms, macrolides, lincosamides, streptogramins B (for Gram-positive bacteria) and fluoroquinolone antibiotics (for Gram-negative bacteria) are unable to bind to the target site of the bacteria, thus making the drugs ineffective to destroy or kill the bacteria [18,38,39]. 

NPs utilise a few modes of entry into the biofilm matrix to disrupt the membrane’s cells for cell lysis or cell death. Metals NPs, such as silver, have an active surface area and undergo oxidation to release the Ag+ ions in which these ions fuse to the surface of the bacterial cell wall. The oxidised silver ions that contain more antibacterial properties will penetrate the cell membrane and destroy the cell, which causes the cell to lose its integrity leading to death. [40]. The carbon NPs will elicit electrostatic charges after encountering bacteria and binding to the external part of the cells. The NPs then enter through the damaged cell, destroying DNA replication and draining out the intracellular contents from the cells [41]. Lipid NPs, such as liposomes with positive charges, could damage the biofilm even at low concentrations and retain the capacity of the drugs before assisting the drugs to penetrate the biofilm to kill the cells [40].

Some powerful tools allow the interaction and penetration of NPs into the biofilm such as surface-sensitive techniques, high-resolution microscopies, and synchrotron-based spectroscopies. One example of a surface-sensitive technique is the use of heat produced from gold NPs localised on the surface plasmon resonance. After gold NPs are irradiated, the photon will be absorbed, reflected, or dissipated based on the physical appearance of the NPs. Plasmon is formed after the visible light or infrared radiation is absorbed. Plasmon then changes to hot electrons, which will be balanced with a lattice and release the energy to the neighbouring area. This energy becomes thermal energy and destroys the bacterial cells and biofilms [42]. The second example is the high-resolution microscope. The pH-sensitive polyacrylamide nanosensor (fluorescent nanosensors) that can pierce biofilm developed by *P. aeruginosa* and *S. mutans* is able to detect the physiologic pH changes in these biofilms in real-time at the microcolony level. The pH level is elevated while the bacteria start to adapt to acidic conditions until the biofilms are well established. The different gradients of pH can be determined within the individual microcolony, either at the core of the microcolonies or at the edge of the colonies. For example, pH 3.5–4 was observed in the core of the microcolonies and pH 5.5–6 was observed at the edge of the colonies for *P. aeruginosa* [43]. One of the synchrotron-based spectroscopies was infrared-attenuated total reflectance (IR-ATR) spectroscopy using silver-metal oxide-Teflon-like (CFx) composites to study the antibacterial activity on *P. fluorescens* biofilm. IR-ATR uses bands to observe biofilm formation or disruption. The bands indicate bacterial adhesion and biofilm expansion. The early stage of the biofilm was observed at the ATR crystal surface followed by EPS and nucleic acids formation. Meanwhile, after modification of the ATR crystal with Ag-CFx, the results demonstrated that in 2 h, the bands related to amide II and EPS were zero, indicating complete elimination of biofilm from the waveguide surface. In contrast to nucleic acids, the bands remained the same throughout the time frame. This could be due to the high antimicrobial activity that is correlated to cell apoptosis and also the reason for the cell membrane destruction of *P. fluorescens* [44].

**Figure 3:**
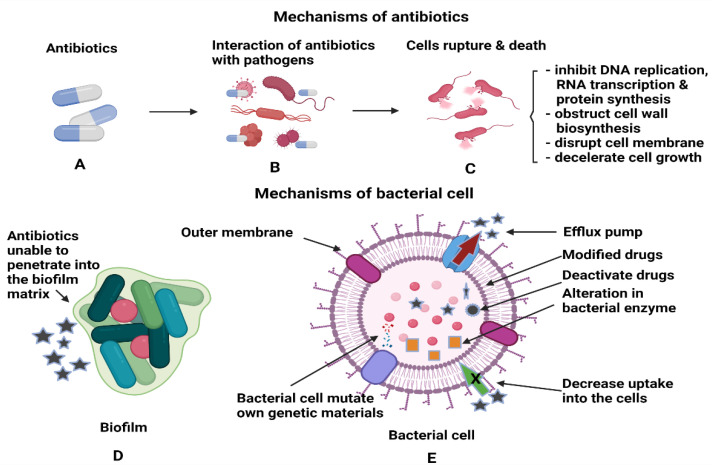
Illustrations of (**A**,**B**) the interactions of antibiotics with the pathogens and (**C**) antibiotics’ activities after penetration into each bacterial cell to kill the cells. (**D**) Antibiotics inefficiency n killing the bacterial cells, as the cells are protected within the biofilm and (**E**) the mechanisms utilised by bacterial cells to render the antibiotics’ functions.

Another type of bacterial enzyme which is commonly found in Gram-negative bacte-ria is plasmid-encoded β-Lactamase namely, TEM, SHV, and CTX-M. These enzymes can reduce the effectiveness of drugs such as penicillin, cephalosporins, monobactams and carbapenems by altering the drug binding sites or reduce uptake of drugs due to the changes in the outer membrane of the bacteria. The inactivation of the drugs is based on the hydrolysis process between water molecules from the enzymes and β-lactam rings of the drugs [45]. Figure 3 shows the interactions between antibiotics and bacterial biofilms and how these can lead to antibiotic resistance. Another method the bacteria employ to prevent the entry of antibiotics to the target site is through the efflux pumps, which are the membrane proteins of bacteria. The efflux pumps will export the drugs from the bacterial cell back to the environment, thus increasing their tolerance to antibiotics. *P. aeruginosa* is an example of bacteria that modulate the transcription regulator of CpxR, which activates efflux pumps and reduces cell membrane permeability to resist drug treatment [46]. Most bacterial species can express efflux pumps either from the same superfamily as a single type of efflux pump or many types from multiple superfamilies [47]. The mechanisms stated above can still operate at the single cell level even after the biofilm formation [48]. The primary functions of antibiotics are to obstruct the essential cellular processes of microbes, specifically, DNA replication, RNA transcription, protein synthesis, impede the synthesis of bacterial cell wall, destruction of cellular membrane, deceleration of cell growth and cell apoptosis [49]. After the formation of biofilm, it is difficult for antimicrobial agents to penetrate deep into the biofilm, and this leads to the drug resistance of bacteria. In addition, bacterial cells that reside deeper in the biofilm tend to multiply slower with lower metabolic activity; hence, they are tolerant to the traditional types of antimicrobial agents [48]. Therefore, coupling NPs with antimicrobial agents could enhance the antimicrobial activity either by piercing into the cell membrane or as drug carriers to directly introduce the NPs to the targeted area for cell disruption [50]. 

## 3. Mechanisms of Actions of Antimicrobial-Loaded Nanoparticles

### 3.1. Carbon-Based Nanoparticles (CBNs)

CBNs, including graphene oxide (GO), graphene quantum dots (GQDs), carbon nanotubes (CNTs) and fullerene, have shown a tremendous impact on the biomedical field with a large contribution to photoluminescence applications, such as fluorescence bioimaging and biosensing, with a high loading capacity to deliver therapeutic molecules [51]. Carbon NPs possess extraordinary optical, mechanical and electrical properties due to the fact of their C–C bonds when they are in heterocyclic conditions that enhance their thermal and electric conductivities, increase high surface area and establish good stability and the ability of the carbon particles to penetrate a potential energy barrier with a height greater than the total energy of the particles [51,52]. These properties are beneficial for microscopic cell and tissue observations for diagnosis and disease treatment, hence, making them promising candidates for therapeutic purposes [53]. 

Each CBN has a different mode of mechanism to produce broad-spectrum antimicrobial effects. In general, electrons in QDs move freely through conduction after exposure to ultraviolet light. The conduction among electrons causes the release of free radicals which, in turn, induces oxidative stress that can destroy the cellular components of the microorganisms. Therefore, QDs produce various kinds of therapeutic effects based on their sizes and structures [54]. A recent paper by Atiqah and co-workers (2021) explained that QDs have more antibacterial activity against Gram-positive (*S. aureus*) bacteria compared to Gram-negative bacteria (*E. coli*). This is because the cell membranes of Gram-negative bacteria are made of lipids, proteins and lipopolysaccharides that are difficult for QDs to destroy. Exposing QDs to light radiation or in an excited state might help QDs to enhance antibacterial properties that kill Gram-negative bacterial cells more efficiently through ROS [55]. In an article by Aliamradni and colleagues (2019), the sizes refer to the thickness of graphene-based NPs and structures either in QDs, platelets, ribbons, and sheets. For example, GO that contains 5–10 sheets of ultrafine graphite and thicknesses less than 100 nm exert antimicrobial properties by activating reactive oxygen species (ROS) to disrupt the biological macromolecules of the microbes [56]. GO and another modified form of GO, known as reduced graphene oxide (rGO), are toxic to Gram-positive and Gram-negative bacteria. Although a study reported that GO caused minor disruption to bacterial cells due to the fact of its negative charge, making it unable to interact with the bacterial cell membrane [57], a contradictory finding stated that bacteria growth can be inhibited if the GO is in a high concentration-dependent manner [58]. In addition to the size and thickness, the antibacterial mechanism of GO entirely depends on the source compounds as well as the oxidation and exfoliation processes [59].

In addition to the ROS, the nanosheets of GO and its derivatives contain hydroxyl, epoxy, and carbonyl functional groups. The sharp edges of the sheets prevent microbial infections when in direct contact with bacteria by breaking down the bacterial cell membrane, decreasing the energy barrier needed for membrane penetration thus leaching out the cell’s contents and, finally, causing bacterial cell death [60]. Besides the sharp edges, another advantage of GO nanosheets are the large surface area that allows it to pierce through the membrane and withdraw phospholipid content from the membrane after GO interacts with the lipid molecules of the bacteria’s cell membranes. A study used varying concentrations of GO nanosheets to observe the effects on different levels of biofilms formed by *S. mutans* that cause dental infections. The findings showed that GO activity was based on time and was dose dependent. As the concentration increased from low to high, the efficacy against the biofilm’s formation increased, and the biomass of the living bacteria decreased within 24 h. This was because GO could inhibit bacterial cell attachment and prevent bacteria from forming biofilm at an earlier stage. However, once the biofilm was completely formed after certain hours, the effects of GO were reduced, and no antibacterial activity was found against the bacterial strains. The study concluded that GO has antibacterial properties only at the early stage of biofilm formation [61]. A similar study was conducted to determine the effects of GO nanosheets against the biofilm of *S. mutans*. The researchers found that GO activity was concentration-dependent, whereby the antibacterial activity was elevated at the lowest concentration (80 μg/mL) with an increase in toxicity due to the high level of functional groups containing oxygen. This study proved that GO nanosheets worked against both planktonic bacteria as well as on the biofilms of *S. mutans* [62]. 

Compared to GO, GQDs have less toxicity to cells. Sun and co-workers (2014) reported that in treating wounds infected by microbes, GQDs could be ideal for incorporation into wound dressings, as they have potent bactericidal effects. However, to have a better performance against infections, GQDs could be treated with H_2_O_2_ cleaved into hydroxyl (OH–) radicals [63]. The application of H2O2 alone as a traditional disinfectant for chronic wounds could induce cytotoxicity to the cells and be less effective as a germicidal agent. Hence, in the OH– form, the antibacterial properties are enhanced and safe for use in wound treatment [64]. Concurrently, combining GQDs and OH– radicals will reduce the side effects of cytotoxicity and impede the formation of biofilms by damaging the intercellular components of the matrix for both Gram-positive bacteria (*S. aureus*) and Gram-negative bacteria (*E. coli*) [63]. Hirschfeld and co-workers (2017) investigated the role of carbon nanotubes against infection by *S. epidermidis* after implanting devices for prosthetic joint infections. Due to the antibiotic resistance and open injury, the bacteria gained access into the body and formed biofilm, which was hard to remove. The investigators modified the CNTs into multi-walled carbon nanotubes (10–200 nm in diameter and 700 nm in length) layered with titanium alloy discs, TiAl6V4, and infused with antibiotic rifampin to test on bacterial cultures. The results revealed that the CNTs retained the antibiotic for a long term with nanoporous titanium surfaces, which allowed the drugs to penetrate and successfully reduce biofilm formation by *S. epidermidis* [65]. Polymethyl methacrylate (PMMA) is widely used in facial surgery and dental implantation. It has good tensile strength and biological characteristics that are suitable for applications, while CNTs possess good antimicrobial properties. The advantage of incorporating CNTs into PMMA could inhibit microbes, such as *S. aureus*, *S. mutans* and *C. albicans*, from adhering to the implanted devices’ surface, thus reducing infections. In brief, the composite can be used directly upon contact with these microbes without impregnating any antibiotics [66]. Fullerene is another carbon-based NP with photochemical activity that generates ROS upon exposure to light, leading to antimicrobial activity, especially in Gram-positive bacteria such as *S. pyogenes* [67]. Furthermore, fullerene damages the cell membrane, disrupts the DNA contents and alters the metabolism pathways to inactivate the microorganisms [68]. 

**Figure 4:**
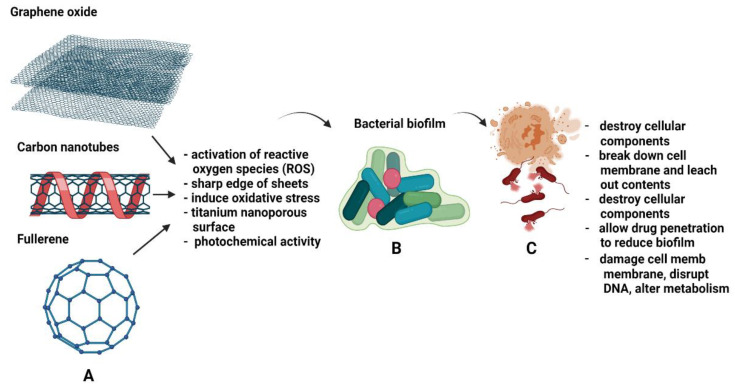
Schematic of (**A**,**B**) the types of carbon-based nanoparticles and mechanisms used by nanoparticles to exert antibacterial activity against biofilms. (**C**) The effects of bacterial cells after nanoparticles penetrate the biofilm.

To have better antimicrobial activity against bacterial strains, some studies utilise more than one type of NP or add other components into the carbon composites. An example is to load carbon NPs with Fe3O4 (iron oxide) to provide more antibacterial effects through agglomeration and total degradation of *E. coli* protein, cell membrane and DNA [56]. For Gram-positive bacteria, especially *S. aureus*, combining GO with Ag metals could prevent cell mitosis, growth or reproduction of bacterial cells [69]. Figure 4 shows examples of CBN in exerting antibacterial effects against bacterial cells and biofilm formation. Further research and clinical trials are necessary to determine the safety and biocompatibility of CBN prior to introducing it to the human body.

### 3.2. Metal-Based Nanoparticles

Antimicrobial drugs prevent bacterial cell wall production and inhibit all the essential functions carried out by the cellular components of bacteria. Due to the fact of resistance, bacteria can overcome these mechanisms by proliferating and agglomerating to form biofilm. Unlike antibiotics, metal-based NPs directly target the cell wall, destroy and damage the DNA structure, inhibit enzyme function and cease the cellular processes of a bacterial cell [70]. Theoretically, there are three common mechanisms utilised by metallic-based NPs to exert their antimicrobial activity. These mechanisms include targeting the bacterial cell membrane (phospholipid bilayer), protein and DNA disruptions and depletion of ATP production and generation of ROS [71]. Briefly, when a positive electron charge of NPs and a negative electron charge of bacteria from a phospholipid bilayer are brought together, there is an elevation in oxidative stress leading to the loss of cell membrane integrity. The overall charge of the membrane is altered, resulting in local membrane disruption and an increase in permeability and, finally, impairment of the cellular function and cell destruction [72]. Due to the physical structural differences between Gram-positive and Gram-negative bacteria, silver NPs have demonstrated greater antibacterial properties against the thin layer of Gram-negative bacteria compared to the thick peptidoglycan outer layer of Gram-positive bacteria [73]. Upon contact with infectious pathogens, the positive charge of silver ions attaches to the negative charge of pathogens’ cell membranes due to the electrostatic force after the oxidation process by silver NPs. In addition, silver NPs also adhere to the bacterial cell walls that have sulphur content protein. Once attached, the cell becomes less rigid, increases its permeability and the cellular components start to leach out from the cells. Silver NPs also perforate into the bacterial cells to damage the intercellular components. Unable to sustain bacterial life processes, the cells almost died and form a hollow shape termed the “ghost cell effect” of the bacterial cells. At this point, the cells are considered dead [74]. 

Metallic NPs induce an antimicrobial response through the protein binding sites of the cells, which could halt the amino acid functions and denature the proteins. Some bacteria mistake metal ions, such as gallium ions (Ga), as their chemical components and utilise them for further bacterial life processes. After uptake of ions into the cell, gallium ions interfere with molecular and biochemical processes that impede the bacterial metabolic system and kills the bacteria [75]. Metallic NPs also kill bacteria by generating ROS, such as hydrogen peroxide or superoxide, that leads to severe oxidative stress, hence, damaging the cells’ molecules, inhibiting enzymes, disrupting the DNA or RNA and obstructing the bacterial biofilm [76,77]. In addition, oxidative stress also forms holes in the bacterial membrane, causing discharge of fluids from the cell. This mechanism can be found in silver NPs modified with curcumin, showing some bactericidal effects against *B. subtilis* and *E. coli*. The bacterial survival rate was 10% after treating the cells with curcumin-based silver NP composites [76]. Metal NPs also exert antimicrobial effects against other types of Gram-positive and Gram-negative bacteria and certain fungi species, namely, *A. niger*, *F. oxysporum* and *A. fumigatus*. Silver attracts more attention than any other metal NP, because it is from plant extracts, especially herbal plants, and components such as vitamins, amino acids, alkaloids, and enzymes. It is also easy to process and contains high toxicity to kill bacterial cells and reduce fungi growth [78]. 

Many antibacterial substances, such as herbal plant extracts, are added with metal NPs to have synergetic effects against microbial infections because they are eco-friendly with better improvement for therapeutic and biocompatibility [79,80]. Ayurvedic herbal plants, such as *Tinospora cordifolia*, are useful for treating numerous types of diseases including diabetes, urinary-associated diseases, uraemia, and lead poisoning. A study was conducted to investigate the role of gold NPs (AuNPs) as a potent antimicrobial agent produced from *T. cordifolia* extracts. The researchers found that *P. aeruginosa* was unable to proliferate or attach to the surfaces and resisted biofilm formation after reacting with AuNPs [81]. A similar study was conducted on *P. aeruginosa* with a similar outcome by conjugating AuNPs with baicalein, a type of Chinese medical plant [82]. These results indicate that these medicinal plants possess remarkable pharmacological properties and could synergistically with NPs become antibiofilm agents in treating chronic infections caused by various pathogenic microorganisms. In addition to antibacterial properties, AuNPs exert antimicrobial effects against HIV, anti-malarial and anti-tumour [81,82].

A study was conducted to examine the effects of different concentrations of silver NPs on bacterial biofilm and EPS matrices formed by the multi-drug resistant *Klebsiella pneumoniae*. The study demonstrated that as the concentration of silver NPs increases, the formation of biofilm decreases due to the EPS matrix being destroyed by the penetration of Ag ions into the cells and blocking biofilm production [83]. Silver NPs were also found to play a role as inhibitory agents to combat urinary tract infections caused by *E. coli*, *Klebsiella* species, *Pseudomonas* species, *Citrobacter* species, coagulase-negative *Staphylococci*, and *Candida* species that were acquired through a medical device such as a urinary catheter. The silver NPs were mixed with the antimicrobial drugs amikacin and nitrofurantoin before being inserted into the mice via a catheter. The study showed that silver NPs eliminated the biofilm from *E. coli*, and by collaborating with the drugs, the efficiency of the drugs increased to tackle the infections and overcome the resistance [84]. Copper NPs (CuNPs) also exhibit a strong ability to suppress microbial colonisation by bacteria, viruses and fungi [85]. Fungi infections are quite common in humans, especially those with low immunity, undergoing antibiotic therapy, surgery and insertion of any medical device into the body based on medical conditions [86,87]. A common fungi infection is by the *Candida* species, mainly *C. albicans*, which is less effective compared to fluconazole. The virulence factors of this fungi species lie between the yeast and hyphae, and this species also secretes enzymes, such as hemolysin, phospholipase and hydrolytic proteases, which are detrimental to immune cells and initiate infections. Unlike bacterial biofilms, *C. albicans* form a well-organised biofilm consisting of yeast, hyphae and pseudohyphae that can resist antibiotics. Rasool and co-workers (2019) reported that CuNPs can diminish the fungi biofilm by targeting the quorum-sensing mechanism of fungi cells that are used for cell-to-cell signalling. CuNPs break this signalling pathway and retard fungi growth, which is more efficient compared to antifungal drugs (i.e., fluconazole) [88].

Some studies showed antibacterial activity using metal oxides for microbial infections. For example, the combination of two metal oxides, zinc and magnesium, into hydroxypropyl methylcellulose (polymer film) showed inhibitory effects on *Proteus mirabilis* biofilm at the lowest concentration of 0.0011% of ZnO:MgO NPs. *P. mirabilis* is a urinary medical-device-associated pathogen. To reduce infections caused by *P. mirabilis*, the polymer film was layered to the urinary catheter to enhance the therapeutic effects by delivering the drugs slowly to the infected sites. Both zinc and magnesium oxides have more excellent antibacterial activity against Gram-positive and Gram-negative bacteria [89]. 

**Figure 5:**
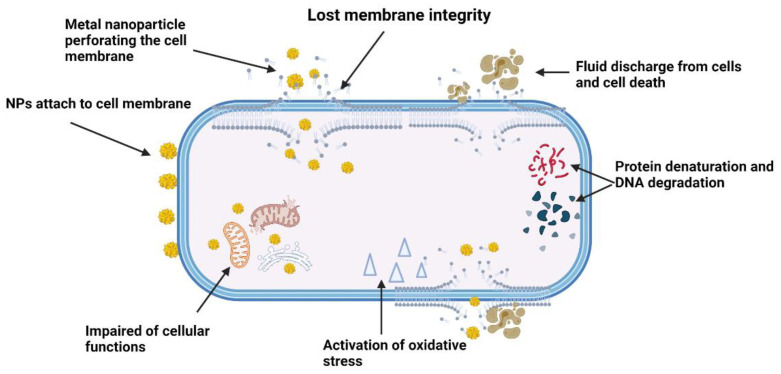
The schematic mechanism of actions of metal nanoparticles on bacterial cell membrane after successfully entering the biofilm matrix.

In another study, nickel NPs showed several antibacterial properties on the clinical isolates of *S. epidermidis*. At the lowest concentration of 0.01 mg/mL, there was a reduction in the formation of biofilm and some haemolytic activities were observed. Although the study reported a non-significant finding, the authors concluded that nickel NPs have the potential to be antibiofilm agents against *S. epidermidis* [90]. Nickel oxide NPs (NiO NPs) that are similar to zinc and magnesium oxide are also being explored for their antibiofilm properties against *P. aeruginosa*. Upon contact with the bacterial cell wall, NiO NPs started to destroy the cell wall by altering the structural membrane of the cell and modifying the replication of protein and DNA to halt the cellular process. This resulted in deceleration of the bacterial growth, leaching out of the contents and finally cell death. Furthermore, NiO NPs also exhibit low toxicity in vivo for *Artemia franciscana* [91]. Figure 5 shows the mechanism of actions of metal NPs against bacterial infections.

### 3.3. Natural Polymeric and Synthetic Polymeric Nanoparticles

**Figure 6:**
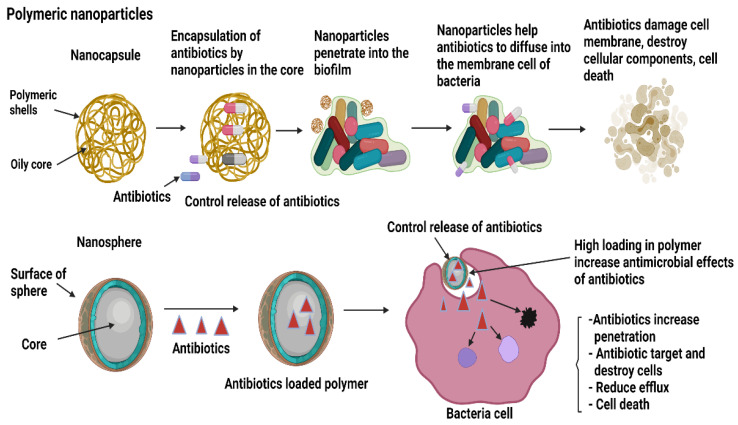
The mechanism of actions of polymeric nanoparticles as drug carriers via controlled release of drugs to target and destroy biofilms.

Fundamentally, polymeric-based NPs have shown great potential for targeted delivery of drugs in the biomedical industry due to the fact of their intriguing properties such as good biocompatibility with host cells, biodegradability, harmfulness to pathogenic microbes, quench toxicity effects and controlled release of drugs for the treatment of several diseases [92]. Polymeric NPs carry drugs or antibiotics based on their distinct morphological features either in nanocapsules or nanospheres. The capsules of polymeric NPs are made of oily cores with polymeric shells to control the movement of the drugs from the capsules to the external environment, while nanospheres either hold the drugs within the spheres or release them to be adsorbed to the outside surface of the spheres [93]. Several factors influence the release of drugs from their polymer NPs such as the types of components and ratio added to the NPs and the interaction between the materials and the processing methods. In general, there are four methods employed by polymer NPs to release drugs. The methods include diffusion, solvent, chemical interaction, and stimulated release. Briefly, diffusion is the release of drugs evenly in the core before diffusing through the membrane pores in which the solvent is based on the osmotic control release to carry the drug load from a lower concentration to a higher concentration into the centre core. Degradation polymers are more favourable compared to the non-degradable, as they degrade in the body after complete eradication of the biofilm. The release of a drug from certain types of polymer NPs is affected by stimuli such as pH, temperature, magnetic attraction and ultrasound [94,95]. Figure 6 shows the mechanism of polymeric NPs during infections. 

Chitosan nanoparticles (CS NPs) are well known as a good vehicle for drug delivery. An in vitro study showed that CS NPs integrated with oxacillin antibiotics and DNase help antibiotics to permeate through the biofilm and kill bacterial communities while enzyme DNase deteriorates the eDNA of the bacteria. The mechanisms enhance the efficiency of the antibiotics and could improve the therapy [96]. Chitosan is a polymer that reacts with a negatively charged mucus to form a substance through a hydrogen bond or hydrophobic bonding. It is known that chitosan tends to aggregate or form clumps from a neutral to higher pH due to the fact of its ability to solubilise in acidic solution or a partial neutralisation process [97]. Chitosan can be modified to alter its properties for better performance as drug carriers such as blending involving the simple mixing of two or more polymers. Examples include mixing chitosan with polyvinyl alcohol to enhance its mechanical and barrier characteristics, chitosan chloride with N-trimethyl for intestinal solubility, thiolated chitosan loaded with NPs to enhance its mucoadhesiveness and grafting carboxylated chitosan with poly(methyl methacrylate) to react with the pH in the environment [98].

Bovine serum albumin nanoparticles (BSA NPs) have emerged as drug carriers for various diseases [99]. Yang and co-workers (2020) incorporated LL-37 with BSA NPs into the biopsy samples of mice infected with *P. aeruginosa* to observe the efficacy of this composite in preventing biofilm formed by *P. aeruginosa*, which is a causative agent of pulmonary infections [100]. LL-37 acts as an antibiofilm against *S. aureus* and *P. aeruginosa*. However, LL-37 is relatively low in stability and toxicity and is degraded or broken down by certain proteolytic enzymes in the human body, such as trypsin, pepsin, elastase, and plasmin or the enzymes aureolysin and V8 protease secreted by *S. aureus* itself, which require a large concentration of drugs to obtain treatment effects [101]. BSA NPs help in stabilising the LL-37 peptide from degradation and control the release of peptides at lower concentrations that can directly target and kill bacterial cells within the biofilm. Furthermore, they block the proliferation of bacterial cells, hence, significantly reducing the secretions of inflammatory cytokines by macrophages, gradually improving lung injury compared to LL-37 alone. These results indicate that BSA NPs are excellent nanocarriers, inhibiting bacterial biofilm, enhancing tolerance to an antibiotic and could become a novel therapy to treat other bacterial species causing pulmonary infections [100].

Recently, Flockton and co-workers (2019) compared surface-modified NPs between polymeric and D-galactose to examine the severity of infections by *P. aeruginosa*. They observed that *P. aeruginosa* induces quorum sensing to activate the degree of pathogenicity, cell attachment, invasion into the surrounding tissue and biofilm production through bacterial cell surface lectins, known as galactose-binding lectin LecA (PA-IL) and fucose-binding lectin LecB (PA-IIL). To weaken these infection mechanisms, LecA, binds to the multiple copies of ligands on the modified surfaces of polymeric NPs. This will inhibit the function of LecA and encapsulation of the drugs into the lipophilic core of the polymeric NPs and control the release of the drugs, which finally diffuse into the biofilm. This destroys the biofilm and reduces the virulence compared to the modified D-galactose NPs (control group) with lower efficacy to both pathogenic factors [102]. Kłodzinska and colleagues (2019) prepared nanogels by combining two NPs of octenyl succinic anhydride-modified HA (OSA-HA) and poly (lactic-co-glycolic acid) (PLGA) that are classified as natural and synthetic polymer NPs, respectively. As drug carriers, OSA-HA encapsulated the peptides of antibiotics, and PLGA disseminated their contents into the biofilm formed by *P. aeruginosa* in the mucus lining of the lungs. They concluded that both NPs served well by diffusing into the mucosal matrix, but only PLGA remained within the biofilm for an extended period. However, when both NPs were combined, they worked excellently to deliver the drugs into the matrix, hence, inhibiting the bacterial growth and reducing the virulence factors in an in vitro model [103]. 

Poly (lactic acid) (PLA) possesses good physical, mechanical and biodegradable properties and is compatible with living tissue or cells, and it has significantly less potential to induce an immune response. In addition, these special features and as a good drug carrier make it widely applicable to be used by conjugating it with other molecules or substances, such as enzymes, chelating agents, peptides, metals and drugs, as additional components during infections to protect from invading pathogens [104]. In a recent study, PLA incorporated with ketoconazole antibiotics was used as NPs to treat fungal infections caused by *Dermatophytes* and *Candida* species on skin, nails, and hair. The in vitro findings revealed that the diameter of PLA NPs was 188.5 nm and encapsulation of the antibiotics was 45.8% ± 2.02%. This study concluded that after encapsulation of ketoconazole by PLA NPs, the drugs had more fungicidal properties and delivered the drugs efficiently into the matrixes to disrupt the biofilm of both fungal species compared to the control groups (only ketoconazole) which had no effect against the bacterial multiplications and biofilms [105]. Similarly, another study used polylactic acid NPs (PLA NPs) and polylactic with glycolic acid nanoparticles (PLGA NPs) each loaded with peptides to examine the in vivo antimicrobial activity against methicillin-resistant *S. aureus*, *E. coli* and *P. aeruginosa* [106]. These synthetic polymer NPs have been approved by the Food and Drug Administration (FDA) to be administered as parenteral route drug carriers while some nanoparticles for clinical translations [106,107]. The investigators found that both composites, PLA NPs and PLGA NPs, have high antimicrobial efficacy against methicillin-resistant *S. aureus*, *E. coli* and *P. aeruginosa*. Moreover, these composites do not exhibit haemolytic behaviour towards red blood cells and are considered biodegradable and biocompatible to cells and tissue [106]. 

A new study explored the use of polyacrylic acid with iron oxide NPs to characterise the properties in inhibiting bacterial growth and biofilm production mainly in medical-related devices and acute or non-healing chronic wounds, as the microbes resist the effects of antibiotics after therapy. The fabricated synthesised polyacrylic iron oxide NPs worked via magnetic force. By supplying the alternating current (AC), the NPs adhered to the bacterial cells, entered the matrix, and released free radicals along with a peroxidase kind of activity to cleave and damage the biofilm. Hypothetically, this method could also be an alternative treatment for antibiotic resistance for various types of infectious agents [108]. 

Polymer micelles have been studied as a drug carrier system, especially to enhance the solubility of hydrophobic drugs, a characteristic of many antibiotics. This is due to the fact of their fine particles sizes, good absorbency, ability to retain solution or substances and lower drug toxicity [109]. In a study, three different types of PEGylated polyurethane micelles with different PEG locations (PEG-g-PU, PEG-b-PU and PEG-c-PU) were incorporated with triclosan, a type of hydrophobic antibiotic to evaluate the antibacterial and antibiofilm effects against *S. aureus*. The results demonstrated that PEG-g-PU-triclosan had the highest potency to eliminate and destroy bacterial strains. The PEG will increase the drug storage time, while PCL (polycaprolactone) helped triclosan become more hydrophilic, controlling the release of the drugs and degrading enzyme lipase upon interaction with bacteria. Moreover, the location of tertiary amine in the PU micelles made it change its surface charges and become positively charged in the acidic environment caused by bacterial cells. The positive charge of PU micelles will enhance the penetration into the biofilm, and the encapsulated triclosan had high antibacterial activity. Once the pH of the PU micelles changed to 5.5, the PCL degrades, bursts with the presence of lipase enzyme, and releases the payloads to destroy the planktonic cells and eliminates the biofilm [110]. Polymer vesicles are also extensively studied as a drug carrier in the biomedical field. Compared to phospholipid vesicles, polymer vesicles are more stable, have good tensile strength and are less permeable. Chen and co-workers fabricated polymer vesicles (TPPBVs) using porphyrin alternating copolymer P(TPP-a-BDE) vesicles and photothermal excitation to evaluate the drug-resistant bacteria and act as a wound disinfectant. They found that TPPBVs had the highest photothermal antibacterial property for *S. aureus* and *E.coli* as well as eradicated the biofilm of *S. aureus* in an in vivo mice model [111].

### 3.4. Lipid-Based Nanoparticles 

Lipid-based nanoparticles (LBNPs) can be classified into a several types, namely, solid lipid nanospheres (SLNs), nanostructured lipid carriers (NLCs), liposomes, niosomes, ethosomes and transfersomes [112,113]. Similar to polymeric NPs, these lipid NPs have gained much popularity in drug delivery systems as they possess good characteristics such as low toxicity effects, carrying biological moieties that are hydrophilic and hydrophobic, easy preparation of sterile formulations as well as a high payload of drugs by extending the time duration of the drug mechanisms and controlled release of the drugs [112]. Different lipid groups, such as monoglycerides, diglycerides and triglycerides, can be used to modify SLNs that are suitable for encapsulating the drugs, as they are highly stable, control the release of the drugs and prevent the drugs from destroying or undergoing degeneration [113]. A study by Anjum and colleagues (2021) used SLNs, anacardic acid (Ana), chitosan and DNase I as matrix materials to treat biofilm formation caused by *S. aureus*. Ana is a derivative of cashew nutshells of *Anacardium occidentale* with high bactericidal activity. However, Ana is limited in clinical practice as it is difficult to dissolve in aqueous solution but dissolves in lipids or other solvents. SLNs obtain additional stability, cell attraction, cell attachment and fuse negatively charged biofilms through chitosan, while DNase degrade the eDNA of *S. aureus*. Therefore, integrating SLNs with these components could cause the NPs to pierce directly into the biofilm, the DNase to break the biofilm matrix to degrade the eDNA while giving entry to SLN–Ana to disperse the contents into the biofilm and destroy the cells efficiently [114]. SLNs are also used to treat biofilms formed by *S. aureus* on prosthetic device drugs by conjugating it with the rifampin (Rif) antibiotic and cis-2 decenoic acid (C2DA). Rif has shown some antimicrobial effects against this species, but it is less effective in killing these species because the β-subunit of the RNA polymerase of the bacterial enzyme causes mutation to the rpo*B* gene of the bacteria in which the antibiotics are the primary target. Once mutated, Rif was unable to detect and kill the bacteria cells as it became resistant. Cis 2-decenoic acid (C2DA) is added to the compound to disseminate the antibiotics into the biofilm. The results showed that the fabricated biocomposite of Rif–C2DA–SLN had a positive charge, disrupted the negatively charged bacterial cell membrane and caused osmotic rupture. SLNs retained Rif antibiotics within the core that caused them to become more potent as antibiofilm agents, hence, increasing the antibiofilm activity once released into the EPS matrix [115]. 

NLCs are a new generation of SLNs that exhibit effects at the local site of an infected region after being administered topically. For example, terbinafine hydrochloric (TH) is a known anti-fungal drug to treat fungal infections but is less water soluble and is lipophilic, making the drug ineffective. By fabricating NLCs with Th into a gel form, the findings demonstrated that TH was encapsulated in the NLCs’ core for more than 24 h, and the drugs were released slowly from the core, disseminated into the lipid matrix to form gel for cell attachment and had more contact time to exert therapeutic effects [116]. Another study conjugated cationic NLCs with oxacillin to evaluate the bacterial property of methicillin-resistant *S. aureus* (MRSA) that causes cutaneous infection. Soyaethyl morpholinium ethosulfate (SME) was layered onto the NLCs to give a positive charge, which later fused to the negative charge of the bacterial cell membrane. This then creates permeability whereby oxacillin from the lipid core of the NLCs will diffuse into the bacterial cells, promote cell lysis, formation of pores on the bacteria surface that causes leakage of ions and molecules causing cell burst. Furthermore, increasing the positive charge of the nanocomposites will increase the electrostatic charges thereby enhancing the lipophilic interaction. This causes greater antimicrobial activities to damage and kill the MRSA cells in the biofilm. Topically applying NLCs with oxacillin reduced the MRSA bacterial colonisation and the skin became intact with better integrity [117].

Liposome is also extensively studied as a drug vehicle in antibiotic therapy. It has antibacterial properties against Gram-positive and Gram-negative bacteria such as *S. pneumoniae* and MRSA. The core of the liposome is hydrophilic, while the external layer is hydrophobic mimicking the biological membrane to bind to the cell membranes of the pathogens [118]. A study was conducted to determine the effects of rifabutin, levofloxacin and vancomycin drugs against biofilm formation by *S. aureus.* The susceptibility tests and MIC results revealed that rifabutin had more antibacterial properties against the planktonic cells and cells within the biofilm compared to the other two antibiotics. However, it needed high doses to induce the effects in vivo and had potential toxicity and resistance. Conjugating liposomes with rifabutin showed higher loading capacity of drugs, and this negatively charged liposome (added DPPG and DPMG) enhances the communication between liposomes and cells. In addition, it also exerts some antimicrobial properties by controlling lipid composition, binding to the bacterial cell wall, and releasing the drugs into the target site for destruction [119]. Table 1 shows more examples of multi-loaded antimicrobial NPs used in in vitro or in vivo animal models.

## 4. Conclusions and Future Perspectives 

Nanotechnology has progressed to address unmet clinical situations especially diseases caused by infectious agents that have become difficult to cure or heal due to the presence of biofilm formations and the inefficiency of antibiotics to eliminate or remove these agents from the infected regions. Persistent antibiotic resistance is quite alarming and poses a serious issue in medical health. The synthesis of NPs from various resources has paved the way for new alternative approaches in developing novel antimicrobial drugs to overcome this issue. NPs exhibit antimicrobial activity by various mechanisms different from each nanoparticle based on the sizes, shapes, properties, morphologies, electric or magnetic charges, surface coatings and additional substances that are conjugated to enhance the antimicrobial effects against the growth of microbes and inhibit biofilm formations. Different properties and characteristics of NPs enable the investigators to invent or design a novel antimicrobial agent that can be used in various clinical applications. Metallic and carbon NPs display potent antimicrobial activity by directly disrupting the biofilms and destroying the cellular components of the bacterial cells. Meanwhile, polymeric NPs act as vehicles to deliver the drugs by penetrating the bacterial cells and diffusing the drug contents to kill the cells. Studies on NPs could shed some light on the prevention, diagnosis, and treatments of infections and control the production of biofilms. Although these studies have explored the bactericidal mechanism of different NPs in in vitro and in vivo models, extensive studies are needed with different microbial species for each NP to examine the effects on the biofilms as well as to evaluate the effectiveness on certain types of bacteria or on most of them including polymicrobial infections. Different studies draw different conclusions based on each model. Some challenges in utilising NPs are the nano-formulations that could significantly limit the usage transition from in vivo to in vivo thereby hindering clinical practice. The challenges are the fast release of drugs before reaching the target area, low concentration of drug retention, adverse effects of systemic toxicity, biosafety, in vivo distribution, biocompatibility, metabolism, and high concentration of drugs to exert the therapeutic effects that could be harmful to other living tissue, while some NPs could enhance the growth of microbes. Although these are the drawbacks associated with NPs, the benefits of NPs in the medical field have displayed a remarkable effect. Therefore, by minimising the cost of production, more studies on the impact of NPs on human cells or tissue, the environment and evaluating the toxicity of the effects of long-term usage will improve this technology for wide-scale use in industrial applications.

## Figures and Tables

**Table 1:** Examples of antimicrobial-loaded nanoparticles/nanocomposites in vivo or in vivo models.

Nanoparticles/Nanocomposites	Targeted Microorganisms	Mode of Actions	Mode of Applications	Loaded with Drugs or Other Composites	References
**Polymeric NPs**					
(a) Polyethylene glycol (PEG) and poly(lactide-co-glycolide) (PLGA)	*S. aureus* and *P. aeruginosa*	Controlled release of drugs and damage to the bacterial cell membrane	In vitro—human red blood cells and cell lines In vivo—zebrafish	Rutin (natural antioxidant), Benzamide (a type of synthetic antibacterial agent)	[120]
(b) PLGA (lactic acid/glycolic acid)	*S. aureus* and *P. aeruginosa*	PLGA with SFX and TAC, high encapsulation of drugs and drug loading, antibacterial activity, reduced lung inflammation, less haemolytic activity, and systemic toxicity	In vitro and in vivo male mice models	Sparfloxacin (SFX) and anti-inflammatory immunosuppressant Tacrolimus (TAC)	[121]
(c) Poly (lactic-co-glycolic acid)–polyethyleneimine (PLGA–PEI)	Methicillin-resistant *S. aureus* (MRSA)	Electrostatic interaction between positive charge of NPs and negative charge of bacterial cell wall allows drug penetration into the cell and control release of drugs by PLGA NPs → maintains enough drugs at the infection site, inhibits bacterial growth and protein synthesis and kills bacterial cells	In vitro and in vivo male mice models	Clindamycin	[122]
**Carbon-Based NPs**					
(a) Carbon quantum dots	*S. aureus*	Antibacterial and antibiofilm activity with rapid healing for wound infections	In vitro—RBC and cell lines In vivo—wounded rats	Injectable hydrogel	[123]
(b) Fullerene	*P. aeruginosa*	Targeting respiratory chain, destroying bacterial cell membrane, direct contact with membrane lipids and diffusing into the cells	Clinical sample from chronic wound	Sulphur	[124]
**Metal-Based NPs**					
(a) Silver NPs	*C. albicans*, *E. coli* and *S. aureus.*	Antibacterial and antibiofilm activity, activation of reactive oxygen species (ROS), direct contact of AgNPs with bacterial proteins, alter DNA replication and destroy cell wall	Surgical silk sutures	-	[125]
(b) Adhesive methacrylated hyaluronan–polyacrylamide (MHA–PAAm) hydrogel with silver nanoparticles (AgNPs)	*S. aureus* and *E. coli*	Hydrogels promote platelet aggregation and tissue granulation, AgNPs as antibacterial agents, polymer hydrogel control release of silver ions at the infection site	Blood sample, wound infection rat models (lung infections)	Gelatin	[126]
(c) Silver NPs	*S. aureus*	AgNPs impede respiratory chain of the pathogens, prevent bacterial adhesion and growth, bind to nucleic acids, membranes and enzymes to cellular intervention	Rabbit model (osteomyelitis) stainless steels implant to the bones	-	[127]
(d) Gold nanoparticles	*N. fowleri* and *B. mandrillari*	Gold-curcumin nanocomposite—enhance amoebicidal activities, ROS activation (damage mitochondrial membrane, cell death, impair DNA synthesis, affect respiratory chain)	Cervical cancer cells	Curcumin	[128]
(e) Manganese dioxide	*E. coli*	Reduce bacterial attachment and growth in implanted silicon, assist immune system to control the infection, block enzymatic reactions, DNA methylation, lesser biofilm formation	In vivo (silicones implant in rat models)	-	[129]
**Lipid-Based NPs**					
(a) Liposome nanocarriers (near infra-red light activated thermosensitive)	*P. aeruginosa*	Increase permeability at high temperature, more drugs are released out from the core, drug absorption directly to EPS matrix, bacteria cell death in biofilm	In vitro and in vivo (local injection at infected sites) of mice models	Tobramycin	[130]
(b) Niosomes nanocarriers	Methicillin-resistant *S. aureus* (MRSA)	Contact release of the drugs, bind to bacterial cell wall, adsorb into the biofilm, drug release from the lipid core into the bacterial cells, high concentration of drugs diffusing into the cells, down regulation of ica*B* gene expression responsible for biofilm formation, and inhibit bacterial growth	Clinical samples	Ciprofloxacin	[131]
(c) Ethosomes nanocarriers	*C. albicans*	Piercing of HAL through lipid membrane, increase in photodynamic activity, loss of membrane integrity, penetration into biofilm, high load of drugs inside the bacterial cells, further prevention of fungi growth, and biofilm formation in mice	In vitro (bacterial cell lines), female mice for topical application	Hexylaminolevulinate (HAL) (photosensitiser) and fluconazole	[132]
(d) Lipid nanoparticles	Methicillin-resistant *S. aureus* (MRSA)	Nanoparticles have cationic charges that break the bacterial cell wall and allow drugs to enter the cell membrane, high antimicrobial activity of nanocomposites, fewer number of neutrophils are detected at the wounded site indicating the nanocomposites have cleared off the bacterial species from the sites	In vivo (mice models infected with surgical wounds)	Rifampicin (NanoRIF)	[133]
(a) Nanocomposites of silver–graphene oxide	*P. acnes*, *A. radicidentis*, *S. epidermidis*, *S. mitis*, and *E. faecalis*	Positive charge Ag ions attracted by the negative charge of the GO surface, elevation of ROS by Ag–GO, interact with other biological molecules in the cell, irreversible oxidative disruption, prevent DNA replication, cell death, and inhibit biofilm formation	Infected teeth model with artificially prepared canals (ex vivo)	-	[134]

## Data Availability

Not applicable.
